# New regulations for animal research – a chance to shine for in silico approaches

**DOI:** 10.1186/s40203-015-0006-1

**Published:** 2015-02-10

**Authors:** Hamid R Noori, Rainer Spanagel

**Affiliations:** Institute of Psychopharmacology, Central Institute of Mental Health, Medical Faculty Mannheim, University of Heidelberg, Mannheim, Germany

The legal landscape for animal experimentation is rapidly changing. Progress in global regulatory frameworks to improve the protection of animals already has significant implications well beyond academic research. Particularly, the European Union Directives Directive 2010/63/EU 2010 on the protection of animals used for scientific purposes, and Cosmetics Directive 76/768/EEC 1976, a complete ban on the use of animals for cosmetic testing in 2013, impose strong constraints on academia and industry. Hence there is a strong need to improve the welfare of animals, fortify the three R’s principles (Replace, Reduce and Refine use of animals) in EU legislation, and provide alternative approaches for animal research and the testing of new cosmetics. The new regulations are by no means an exclusive European affaire, as they have been reinforced globally by bi- and multi-lateral cooperations.

Therefore, these new regulations will ultimately reshape our way of thinking about basic and preclinical experimental design, by not only limiting the sample size of experiments, but also adding critical restrictions on procedures that require alternative, quantitatively-defined frameworks for experimental setups and analyses. While the three R’s challenge traditional concepts in animal studies, which equate an increase in sample size with an improvement in the robustness of experimental results, they provide a great chance for in silico approaches (Noori and Spanagel 2013) to shine.

Ligand- and structure-based virtual screening procedures for drug discovery (Rester 2008), computational pharmacokinetics (Bois 2013), in silico screening procedures to investigate systemic drug effects (Noori and Jäger 2010; Noori et al. 2012a), mathematically-based optimal experimental design (Fedorov et al. 2002) and novel biostatistical approaches (Beery and Zucker 2011; Noori et al. 2012b) not only bear the potential to improve animal experiments, but can also reduce the number of animals used by eliminating unnecessary testing (e.g., via meta-analysis studies).

In Silico Pharmacology provides an optimal electronic platform for studies in these fields, and through emphasis on the three R’s intends to assist animal researchers in overcoming present and future challenges, contributing to a modernization of their research concepts. In particular, by being indexed in the largest database of biomedical articles established by the US National Library of Medicine (PubmedCentral: http://www.ncbi.nlm.nih.gov/pmc/journals/2586/), it has begun to bring its computer-based publications closer to end-user experimentalist readership and facilitate communication in the interface area of dry- and wet-laboratories.
